# Thirty-day hospital re-admission for Medicaid enrollees with schizophrenia: the role of patient comorbidity and local health-care systems

**DOI:** 10.1186/1940-0640-10-S1-A6

**Published:** 2015-02-20

**Authors:** Alisa B Busch, Arnold M Epstein, Thomas G McGuire, Sharon-Lise T Normand, Richard G Frank

**Affiliations:** 1 Health Services Research Division of Partners Psychiatry & Mental Health, McLean Hospital, Belmont, MA, USA; 2 Department of Health Care Policy, Harvard Medical School, Boston, MA, 02115, USA; 3 Department of Health Policy and Management, Harvard School of Public Health, Boston, MA, 02115, USA; 4 Division of General Medicine, Brigham and Women’s Hospital, Boston, MA, 02115, USA; 5 Department of Biostatistics, Harvard School of Public Health, Boston, MA, 02115, USA; 6 National Bureau of Economic Research, Cambridge, MA, 02138, USA

## Background

Early re-admission after hospitalization is an increasing focus of health-care policy because it results in high costs and often reflects opportunities for improving treatment quality. The goal of this study is to examine the relationship between 30-day mental health/substance use disorder hospital re-admission for persons with schizophrenia, and patient characteristics, hospital utilization, and community treatment quality and capacity.

## Materials and methods

Observational study of schizophrenia-diagnosed enrollees having ≥ 1 behavioral health hospitalization in 2005 from 18 state Medicaid programs (N = 28083). Regression models examined the relationship between 30-day behavioral health hospital re-admission, enrollee characteristics (demographic and comorbidity), and county-level indicators for: 1) quality of care (antipsychotic and behavioral health visit continuity, behavioral health visit within 7 days post-hospitalization); 2) behavioral health hospitalization (length of stay, admission rates); and 3) treatment capacity (e.g., population-based estimates of outpatient providers/clinics).

## Results

Fifty-one percent of the study population had a co-occurring substance use disorder; nearly 47 percent had a co-occurring chronic general medical condition. Enrollee comorbidity was associated with higher predicted probability of 30-day behavioral health re-admission, particularly for enrollees with substance use disorders (predicted probability [95% CI] = 23.9% [21.5%–26.3%]) versus without (14.7% [13.9%–15.4%]). Chronic medical conditions were associated with increased re-admissions in a dose-response manner (e.g., ≥ three: 25.1% [22.1%–28.2%] versus none: 17.7% [16.3%–19.1]). Higher county rate of behavioral health visits within 7 days post-hospitalization was associated with lower re-admission for individual enrollees (e.g., for county rates of 7-day follow-up of 55% versus 85%, re-admission predicted probability = 16.1% [15.8%–16.4%] versus 13.3% [12.9%–13.6%]). In contrast, higher county rate of behavioral health hospitalization was associated with higher re-admission probability for individual enrollees (e.g., for country admission rates 10% versus 30%, re-admission predicted probability for an individual = 11.3% [11.0%–11.6%] versus 16.7% [16.4%–17.0%]).

## Conclusions

Efforts to reduce 30-day psychiatric re-admissions should focus on comorbid substance use and general medical care coordination, as well as factors that contribute to hospitalization in general and improving transitions to community care. Comorbid substance use disorders were particularly prominent in 30-day behavioral health re-admission—patients with comorbid substance use disorders had a 63.7 percent higher predicted probability of 30-day re-admission compared to those without. These findings demonstrate the substantial role of comorbid substance use disorders in behavioral health 30-day re-admissions. They also highlight an opportunity for Medicaid policy to influence improved access to substance use disorder treatment, including its coordination with behavioral health and general medical care, in an effort to reduce 30-day re-admission for individuals with severe mental illness.

